# Factors influencing men to discontinue antiretroviral at Seventh Day Adventist clinic, Maseru, Lesotho

**DOI:** 10.3389/fpubh.2026.1796236

**Published:** 2026-06-11

**Authors:** Thabelang Rabaholo, Moganavelli Reddy

**Affiliations:** College of Nursing and Public Health, University of KwaZulu-Natal, Durban, South Africa

**Keywords:** adherence, art, ARV’S, discontinue, HIV, men

## Abstract

**Background:**

Discontinuation of ARV is a worldwide problem, with a discontinuation rate of 20% globally and 40–50% in Sub-Saharan Africa. In Lesotho, it ranges from 20 to 30% in men. To effectively tackle the HIV/AIDS epidemic in Lesotho, it is necessary to address the several complex problems that hinder adherence to ARVs among men.

**Methods:**

This study used a qualitative descriptive case study approach to collect data from men aged 18 years and above receiving health services at the Seventh-day Adventist clinic, using a homogeneous purposive sampling method. In-depth interviews were used to collect audio-recorded data after obtaining informed consent and 10 men who were on ART were interviewed.

**Results:**

Of the 10 clients, all had discontinued their ARVS in the past year (2023). Overall, participants identified four factors that influence most men to default on their ARVs: individual, socio-economic, culturally related, and health systems factors.

**Conclusion:**

Participant’s difficulties, such as socio-economic obstacles (e.g., transportation costs and unstable employment), cultural influences based on religious and Christian norms that discourage clinic attendance, and deficiencies in the health system (e.g., lengthy wait times and rigid services) were identified by participants as interrelated factors driving men’s discontinuation of ART treatment. These results necessitate that health systems take immediate, comprehensive actions, such as prioritising male-specific ART delivery modalities.

## Introduction

Antiretroviral therapy (ART) has significantly improved Human immunodeficiency virus (HIV) and Acquired Immunodeficiency Syndrome (AIDS) treatment and patient survival rates globally (1). However, adherence remains a challenge, particularly among men, who have higher rates of discontinuing ART than women, posing a significant obstacle to optimal treatment outcomes (2). In 2024, there were over 40.8 million HIV-positive individuals worldwide, the most significant amount ever. The anticipated 1.3 million new HIV infections in 2024 were only marginally lower than the estimated 1.4 million new infections in 2023. Sub-Saharan Africa accounted for half of all HIV-positive individuals in 2024. Sub-Saharan Africa was home to half of the 9.2 million people globally in 2024 who needed but were not receiving HIV treatment. Approximately 31.6 million persons worldwide, or 77% of all HIV-positive individuals, were undergoing treatment in 2024. By 2025, certain regions had almost reached the 95–95–95 testing and treatment goals. At least seven nations, Botswana, Eswatini, Lesotho, Namibia, Rwanda, Zambia, and Zimbabwe met their 95–95-95 goals (3). The average lost-to-follow-up estimate worldwide is 20%, which is higher than the WHO’s aim of less than 15%, and in sub-Saharan Africa, attrition (death or dropout) exceeds 40 to50% within 2 years of ART initiation (4). Men are less inclined to initiate and maintain antiretroviral therapy until the disease has progressed, complicating efforts to achieve high rates of viral suppression and complete immunological restoration in this population. Male HIV-related fatalities frequently arise from men’s failure to seek HIV treatment or from postponing access to treatment. HIV treatment and care programs disproportionately benefit women and girls over men and boys, as men with HIV experience less favourable health outcomes and are less likely to participate in HIV treatment and care services compared to women (5). Unlike men, women are more likely to receive a diagnosis and start receiving care because of routine testing in settings such as prenatal, antenatal, and postnatal care facilities (6). The idea that the AIDS epidemic can be eradicated as a global public health threat by 2030 is the result of significant advancements in HIV preventive research (7).

HIV is one of the significant public health issues, and it is more prevalent in low- and middle-income countries. Africa is the most affected continent, accounting for about two-thirds of all new HIV infections. Approximately 31.6 (77%) million persons worldwide, of all HIV-positive individuals, were receiving treatment in 2024. By 2025, certain African regions were on the verge of meeting the 95–95–95 testing and treatment goals (3). HIV continues to spread persistently, and morbidity continues to increase in many nations and regions around the world despite significant advancements in our understanding of the epidemiology of HIV infection over the past 20 years and improved access to Antiretroviral (ARV). Understanding the fundamental contributing causes and the social and economic circumstances that promote the spread of HIV needs a great deal more work (8, 9). HIV programs are significantly impacted by socio-economic factors in low- and middle-income nations, such as Lesotho.

Lesotho, a mountainous country in Southern Africa, has an HIV prevalence of 22.7% as of 2022, with women having a much higher prevalence (27.4%) than men (17.8%) (10). Lesotho adopted the “Test and Treat” guidelines in 2016, making all HIV-positive individuals eligible for ART regardless of their CD4 count, and became the first SSA nation to adopt these WHO recommendations (11).

However, a substantial proportion of patients continue to present or re-present for HIV treatment with advanced HIV disease (AHD), with more than 50% of these being male (5). This is a concerning trend, as men have been found to test less frequently than women and report higher levels of anticipated stigma (6). There are a lot of structural economic factors that can affect men’s disengagement from their HIV treatment journey.

Various factors contribute to the high rates of male disengagement from ART in Lesotho. These include work-related issues, fear of disclosing their HIV status to partners, adherence challenges due to traditional beliefs, and insufficient counselling (12). Additionally, socio-economic factors such as poverty and limited access to healthcare services can hinder men’s ability to adhere to ART consistently (13). Health system factors, such as inadequate infrastructure and healthcare delivery systems, may also contribute to men discontinuing treatment (14).

Due to men’s considerable mobility, the HIV program faced challenges in keeping men, resulting in a significant percentage of dropouts from the care and treatment process (15), despite efforts to enhance the quality of care and treatment. From October 2018 to June 2020, 33,398 patients in 10 Lesotho districts were lost to care, with an estimated retention rate of 88% due to insufficient data (16).

Gender stereotypes are the key challenges in influencing men to discontinue HIV treatment. The study conducted in Cape Town, South Africa, indicates that men may experience apprehension due to the potential consequences of social exclusion or negative evaluation if their HIV status becomes public. The negative perception associated with this stigma can result in men intentionally avoiding healthcare treatments, such as ARV treatment, to prevent the exposure of their HIV status (17, 18). In addition, men are typically the primary “breadwinners,” meaning they must work longer hours and travel farther from home. Men may thus have less time to receive HIV care, less experience, and greater discomfort navigating facility-based barriers to care as a result of gendered health facilities and societal obligations (19). Gender norms and cultural practices play a vital role in influencing men to discontinue on ARVs Conventional gender norms frequently prescribe that men should exhibit strength, emotional resilience, and independence. Seeking medical care, particularly for an illness such as HIV/AIDS, might be seen as an indication of fragility or susceptibility. This can discourage males from obtaining and adhering to ARV treatment (17, 20).

The study aimed to explore factors influencing men’s discontinuation of ARVs at the Maseru Seventh-day Adventist Clinic, Lesotho.

## Research methods and design

### Study design

This study used a qualitative descriptive case study design, with qualitative data collection methods, to understand the socio-economic, cultural, and health system factors that influence men’s decision to discontinue Antiretrovirals at the Maseru Seventh-day Adventist Clinic in Maseru, Lesotho.

### Study setting

The study was conducted at the Maseru Seventh-day Adventist Clinic, 5 km from the central business district. The facility is a high-volume facility serving 23 villages surrounding it. The clinic was selected because of the high number of male patients who discontinue treatment even though this is a specialised clinic for men. The facility is currently providing ART to 4,500 patients.

### Study population and sampling strategy

The recruitment was male clients living with HIV attending the Maseru Seventh Day Adventist clinic Men’s clinic who were above 18 years of age with a history of stopping the HIV care program for a minimum of 3 months in the past year and being documented in the Lesotho tracking register. In that register, only 10 male participants were eligible to participate in the study. The researcher chose men only to understand why men are still discontinuing ART services despite having a specialised clinic. The researcher recruited clients with the assistance of Maseru Seventh Day Clinic staff, and those who met the criteria were called for one-on-one interviews at their convenience. The study applied a homogeneous purposive sampling (21). Strategy to understand the socio-economic, health system and cultural factors influencing men to discontinue their ARVs. For this reason, 10 participants were interviewed for the research.

### The inclusion criteria

The inclusion criteria were based on recruiting male HIV-positive clients who were attending clinics at the Maseru Seventh Day Adventist clinic with the following characteristics: In the past year, they had discontinued on treatment and appeared in the Lesotho tracking register, male clients above 18 years of age and able to provide informed consent.

### The exclusion criteria

The exclusion criteria were HIV-negative males and females receiving services at the Maseru Seventh Day Adventist clinic and all male HIV-positive clients who had not defaulted on treatment in the past year.

### Data collection

After receiving approval from the Biomedical Research Ethics Committee, the Ministry of Health Research Ethics Committee, and the District Health Management team in Maseru to access the study facility, data collection commenced on 11 December 2024. Before the interview, the researcher explained the topic thoroughly to the participants and obtained informed consent to participate in the study. To assess the adequacy of the research interview guide questions, the researcher selected a single participant at the Domiciliary Clinic in Maseru, 5 km from the Maseru SDA clinic. After this pilot study, the interview guide was reviewed, and additional questions were added.

The researcher conducted in-depth, face-to-face interviews using an interview guide.

An interview guide and a reliable audio recorder were used to capture the interviews (Consent was obtained to record them). This allowed the researcher to monitor non-verbal behaviour. An in-depth interview is a rigorous, exploratory approach to obtaining comprehensive information from an individual (22). The researcher conducted the interviews in Sesotho in a private consultation room. The translator was not used during the interview as the researcher spoke his native language, and the interview guide was also in Sesotho. The data collection process was terminated on the 10th participant due to redundancy and data saturation.

### Data analysis

All the interviews were recorded and transcribed verbatim into sesotho, after which the researcher translated them into english and then back-translated them to ensure accuracy. The sesotho transcripts were shared with all 10 participants for data verification. The researcher conducted manual thematic analysis (23). After data collection, the researcher followed six steps of thematic analysis, during which he familiarised himself with the gathered data. Then he selected keywords from the transcriptions to identify patterns. Codes were not predetermined but rather emerged from the data, and about 600 short phrases and codes were extracted from 10 transcriptions. The categorisation of codes yielded 60 categories. About four themes emerged with eleven subthemes. Basic demographic details of participants were collected.

### Trustworthiness

To ensure trustworthiness, prolonged engagements and interactions were conducted with the participants during the interviews. Data collection utilised audio recordings to enhance accuracy of the data. During data analysis, data cleaning and member checks were performed. Audio recordings and field notes were compared with the interpreted data to assess, correct errors and seek additional information as needed. A reflexive journal was utilised in which the researcher recorded their preconceived notions about the research topic.

### Ethical considerations

The Biomedical Research Ethics Committee (BREC) of the University of KwaZulu-Natal (UKZN) (reference no: BREC/00007722/2024) and the Ministry of Health Research Ethics Committee Lesotho (reference no: ID273-2024) granted ethical permission for the study. All participants provided written, voluntary informed consent. Data was reported using pseudonyms to maintain confidentiality.

## Results

### Demographic characteristics of study participants

Table 1 shows the demographic characteristics of the study participants.

**Table 1:** Demographics of participants.

Characteristics	Number	Percentages
Ages (years)		
30–34	6	60%
35–39	1	10%
40–44	2	20%
45–49	0	-
50–55	1	10%
Education level		
Primary	2	20%
High School	7	70%
Tertiary	1	10%
Marital status		
Single	2	20%
Married	8	80%
Separated	0	–
Divorced	0	–
Employment status		
Employed	4	30%
Self-employed	5	60%
Unemployment	1	10%

The researcher interviewed a total of 10 eligible male ART clients. Of the 10 clients, all had discontinued their ARV in the past year. The participants’ ages ranged from 30 to 50 years, with a median of 34 years. Most Participants were self-employed, four were employed, and one was out of work. Seven participants in this study had completed high school, 2 had completed primary school, and 1 had a tertiary diploma. 80% (8/10) of the participants in the study were married, and the remaining 20% (2/10) were single Table 2 shows The participants identification numbers and their duration on ART treatment.

**Table 2:** The participants identification numbers and their duration on ART treatment.

Participant identification	Participant age	Duration of ART treatment
PID-001	50 years	More than 5 years
PID-002	32 years	5 years
PID-003	33 years	4 years
PID-004	31 years	7 years
PID-005	34 years	2 years
PID-006	42 years	4 years
PID-007	34 years	More than 10 years
PID-008	35 years	4 years
PID-009	42 years	8 years
PID-0010	31 years	6 years

### Key themes

The researcher’s data had four themes: interpersonal/individual, socio-economic, cultural, and health systems factors. The participant’s employment status and healthcare workers’ attitudes were key themes. The verbatim codes were pseudonymised using participants numbers to maintain confidentiality (see Table 3).

**Table 3:** Themes and subthemes.

Themes	Subthemes
Personal/ interpersonal factors that influence men to discontinue on ARVs	Denial and unwillingness to accept the HIV status Failure to disclose the HIV status ARV adverse effects or side effects
Socio-economic factors that influence men to discontinue on ARV	Employment status Transport money to the appointment
Spirituality factors that influence men to discontinue on ARVs	Christianity Other socio-cultural practises
Health systems that influence men to discontinue on ARVs	Healthcare worker inefficiencies Facility-related inefficiencies Healthcare worker’s attitudes

### Theme 1: Interpersonal factors that influence men to discontinue on ARVs

The research findings revealed that most participants were unwilling to accept their HIV status, and those who did accept their HIV status did not want to disclose their HIV status to their partners, family or friends. The other finding was that most participants experienced some ARV side effects, which led to disengagement from treatment.

#### Subtheme 1.1: Denial and unwillingness to accept the HIV status

The inability to believe or to admit one’s HIV status or failure to confront the reality of living with a chronic disease. Some of the participants explained that the main influence was their inability to accept that they were living with HIV. So, it was difficult for them to access ARVs and blood draw check-ups at the facility due to the acceptance of their HIV status. Also, clients lacked knowledge of basic HIV information to understand how they contracted HIV; this gap actually led them to deny their HIV status.

This is indicated in the following responses.

*“The health care worker told me of this facility that I’m HIV positive and wanted to counsel me. I did not believe what she told me because I know my HIV negative status, and I told her that if I’m HIV positive, I’m agreeing because I believe that they are telling me the truth. Also, they said they are going to give me ARVs*”*(PID-001).*

*“After receiving those positive results from the lab after 3 months, I did not tell anyone at home because I was busy asking myself how I got infected with HIV. Sir, it takes an act of courage to disclose the HIV status” (PID-002).*

PID-001 further illustrated that he did not believe he was HIV positive, as illustrated in the following quote.

*“I now don’t believe I’m HIV positive, the reason being I’m always drowsy, and I hate being in that state. I’m unable to use my mind” (PID-001).*

#### Subtheme 1.2: Failure to disclose the HIV status

It is when the participant who is living with HIV either for some years or sometimes to inform people around them about their HIV status. Fear of disclosing one’s status was the main influence for men to discontinue on ARVs. Participants did not want their family and friends to know their HIV status. The researcher also found that it was the participant’s sole responsibility to collect their medications and understand the medication’s dates, as accounted for in the following responses:

*“Also, it was difficult to disclose my HIV status to my partner as I’m still in pain about how I was infected, but after informing her about my HIV, I felt at ease” (PID-003).*

*During the past years of my high school, I used to reside at a boarding school; they did not know that I was HIV positive; I had to manoeuvre my way around taking my medications on my own time. As for that, I have never felt discriminated against by my schoolmates” (PID-0010).*

*“I have kept it very private; it’s only my family who knows my HIV status. The only person who has discriminated against me is the health care worker” (PID-002).*

#### Subtheme 1.3: ARVs adverse effects

These are common side effects that people living with HIV experience post initiation on HIV treatment, common symptoms are nausea, diarrhoea, fatigue and sleeping difficulties. Participants in the study reported that discontinuing ARVs also occurred after experiencing ARV side effects, especially in the first 6 months post-ART initiation. They further detailed that they also came to the facility to report the side effects, and the clinician dismissed them and told them it would fade with time. The most reported side effects in the study were vomiting, diarrhoea and drowsiness. As accounted for by the participants in the following responses.

*“After being told about my HIV status and being initiated on ARVs, I was bedridden for about six months due to diarrhoea and vomiting from these ARVs of theirs” (PID-003).*

*“The other day, I advised myself to say I can’t always be drowsy, and these ARVs are poisonous. So, you see, in the morning, I take them and become drowsy the whole day; in the evening, I take them again and become drowsy the whole night. I noticed that I will not have time for planning because I will always be drowsy” (PID-001).*

### Theme 2: Socio-economic factors that influence men to discontinue on ARVs

The research found that participants failed to avail themselves of their ARV check due to work conditions and finances. Some participants explained that their employees were using a “no work, no pay policy,” and those willing to go to the facility did not have the money to access it.

#### Subtheme 2.1: Employment status

Failure to secure the employment status. Most participants highlighted that they do discontinue on medication due to seeking employment in South Africa or due to their strict employers who do not allow them to seek health services.

Participants highlighted that they stopped taking their ARVs mainly because of work-related issues, as some worked outside the country. Those in the country were placed in districts far from Maseru and were uneasy coming for their medication check-up. Those in South Africa also highlighted that coming home to collect their medications was impossible. This is demonstrated below by the research participant’s responses:

*“I stopped coming for my check-up because I was in South Africa” (PID-004).*

*“It was because of the job; as I mentioned earlier, my boss does not allow me to come to the facility because he uses a no-work, no-pay policy” (PID-005).*

*“It is because of my job, because I come by schedule, not anytime I want, so my work schedule conflicts with my clinic appointment. So, it was difficult to come and collect the ARVs” (PID-006).*

*“My challenge was moving from South Africa to Namibia because of work. I had to be there for a year, and at that time, I could not come to South Africa. So, there was no one to help me get medications (PID-007).*

Surprisingly, self-employed participants also mentioned that they stopped taking their medication because they were not able to come to the facility due to work-related issues. This is apparent in the following response:

*“As I mentioned earlier, it is the kind of job I’m doing; I’m a taxi driver, so I’m always busy going for check-ups” (PID-003).*

#### Subtheme 2.2: Transport money to the appointment

Inability for participants to secure transport money to seek services at the health facility. Some participants mentioned that the cost of transport to the health facility had influenced them to discontinue ARVs. Participants mentioned being a bit far from the facility and needing taxi fare for their medical check-ups, given that most were in South Africa and only came after a specific period. This is reflected in the following responses:

*“I did not end up coming home due to unforeseen things; they killed my boss, and I ended up not having money for anything. The main issue was that I did not have transport money” (PID-008).*

*“No support I’m receiving from anyone, I am the one who does everything for me, when medications are concerned, hence most of the time I can’t even afford a 12 Rands taxi fare to collect my ARVs” (PID-003).*

### Theme 3: Spirituality factors that influence men to discontinue on ARVs

The research found that some participants held cultural beliefs and Christianity; others discontinued due to their Christian leaders and cultural norms, such as beliefs in ancestral processes.

#### Subtheme 3.1: Christianity

Christianity and religion, the process of understanding charismatic teaching to follow Jesus.

Participants stated that Christianity and religion played a vital role in their lives and in the community. Some participants believed in religious support, which influenced how they adhered to their medications and appointments. Participants also stated that some charismatic leaders influenced them to stop taking their medications after praying for them. This is demonstrated below:

*“I believe church is extremely important; if a pastor advised me to stop treatment, I would do so without any hesitation” (PID-001).*

*“That’s very true, a lot of men have stopped taking their ARV’S due to some churches, especially charismatic churches, because they say they heal everything, including HIV” (PID-004).*

*“Yes, sir, it is possible; I once met someone who said to me that if I can pray a certain way for a certain period, there will be no need for me to come for a check-up as I will be healed from HIV” (PID-009).*

#### Subtheme 3.2: Other socio-cultural practises (traditional medicines)

Taking unprescribed medicinal products to use with the intention of treating HIV.

Participants noted that cultural beliefs and norms played a vital role in their lives and in the community. Some people believed in cultural norms, while others believed in traditional medicinal products. They further explained that their cultural beliefs and norms influenced them to discontinue their ARVs and to avoid attending appointments. They also stated that some cultural and traditional leaders were so influential in controlling people to stop taking their medications and adhering to clinic appointments. This is demonstrated in the following responses:

*“Well, ancestral or traditional beliefs can lead us to healers who give us false information or say that they can provide us with herbs that cure HIV (PID-002).*

*“It depends on what sense because you might find people who believe that herbal medicines and herbal food are more powerful than taking western medicines, so you find that they may also contribute” (PID-0010).*

*“Yes, many men, especially in the highlands, stopped taking their medications because they use traditional herbs” (PID-004).*

### Theme 4: Health systems that influence men to discontinue on ARVs

This research showed that most participants were not engaging well with facilities due to health worker inefficiencies, such as failing to start the services on time. Another issue was failing to show the patient flow, and the other challenge was the unpleasant attitudes of the healthcare workers when participants came for medication pick-up.

#### Subtheme 4.1: Healthcare worker inefficiencies

The main causes of inefficiency among healthcare workers are severe personnel shortages, enormous administrative obligations, and inadequate digital infrastructure.

Participants stated that one of the reasons they discontinued ARVs was healthcare workers’ inability to arrange their workspace in a timely and cohesive manner, to tell them what to do, and to provide client-centred services. For example, some participants discontinued on ARVs because the healthcare worker did not communicate the next check-up with clients to agree on the preferred time to pick up their medication and to draw blood. They explained further that, in some instances, when the client could not come, they would send someone else, but no medications would be issued to their treatment supporters, which then resulted in them disengaging. The other inefficiency caused by health workers was the failure to manage the biomarker queue, which led to clients spending more time at the facility, as detailed in the following responses.

*“There were times when I used to give her my patient card to collect medication on my behalf until they told her that for my next appointment, she should not bother to come as I’m due for drawing blood” (PID-003).*

*“So far, the services are still good, and they need to improve at the blood drawing department as we take most of our time there” (PID-006).*

Some participants also mentioned that when they planned their trips, they informed their clinicians, especially when they were going to South Africa, but clinicians denied them multi-month dispensing, which led to discontinuing ARVs.

*“I told my clinician that I had found a job in South Africa and learnt that some patients get a 6-month supply of ARVs. I requested the same, but the clinicians told me that I do not qualify for such” (PID-004).*

#### Subtheme 4.2: Facility-related inefficiencies: the facility protocols or processes that cause delays in receiving health services, such as long queues

Some participants mentioned that during the COVID-19 era, they were not allowed to enter the facility premises without a COVID-19 vaccination card. Also, due to the long waiting period at the facility, they had discontinued treatment. The participants explain further:

*“At some point, I remember during Covid 19 times, unvaccinated clients were not allowed to enter facility premises; it was the first time I defaulted on treatment, then came later to explain to them why I’m not vaccinated” (PID-0010).*

*“I think services can be improved by reducing the clinic waiting period. The healthcare workers should stop wasting time by being busy chit-chatting with their colleagues” (PID-001).*

#### Subtheme 4.3: Healthcare worker’s attitudes

Most participants in the study stated that their poor relationship with their healthcare workers was the primary factor in their disengagement from the facility. As accounted for in the following responses.

*“Also, the attitude and approach of some staff have to change; they should stop shouting at us like we are children” (PID-001).*

*“There are no reasons that are facility-related; they just need to work on their attitudes because some staff members don’t communicate with us well” (PID-006).*

*“The bad staff attitudes, they treat us worse than children. They feel like we are under their control. They talk to us in a very discriminatory way, making one want to retaliate. At the time, you feel that it is better not to come and focus on working hard to provide for your family in case someone dies” (PID-002).*

*“The biggest challenge at this facility sometimes is the unfriendliness of the staff or the attitudes” (PID-003).*

*“The main reason men stop coming to get their ARVs is because of the bad attitudes of the clinic staff, and some men end up having a self-conversation that they will never come to the health facility anymore, not to disturb staff at their workplace” (PID-004).*

## Discussion

Factors that influenced men to discontinue on ARVs in this study included interpersonal, socio-economic, and healthcare system-related factors. Personal or interpersonal factors included denial and unwillingness to accept HIV status, failure to disclose one’s status and ARV side effects. The socio-economic factors included employment status and the cost of transport to the appointment. Health system-related factors are healthcare worker inefficiencies, facility-related inefficiencies and healthcare workers’ attitudes. The key findings from this study are employment status, financial burden associated with transport to the facility, and negative attitudes from healthcare workers.

The findings of this study found that most men discontinued their ARVs due to fear of disclosing their HIV status to their partners, family and friends. These findings are similar to those of studies conducted in Limpopo, the Western and Eastern Cape of South Africa (24–27). Studies conducted in Eswatini and Malawi, respectively, found that clients did not share their HIV status with their partners and that clients do not prefer to share their HIV status with their partners (28, 29). A study conducted in Limpopo, South Africa, outlines that clients fear disclosing their HIV status due to perceived stigma and discrimination and fear of rejection by partners and family members (25). Our findings also show that men fear disclosing due to stigma and fear of rejection.

A study conducted in Malawi stated that healthcare workers need to facilitate disclosure through a mutual disclosure agreement. Also, in different countries, they need to strengthen some interventions that target stigma and discrimination for people living with HIV to allow a better disclosure process (29).

This study found that transport costs to the health facility had influenced men to discontinue ARVs The finding are similar to the studies conducted in South Africa, Malawi, Eswatani and Uganda maintained that financial instability or burden faced by HIV-positive persons leads to discontinuing their ARVs as the studies reported that some clients missed their appointments due to lack of transport money to the clinic (24, 28–30). The study’s findings showed that some men disengaged due to transport costs and being far from the clinic, which forced them to seek employment in other parts of Lesotho and in South Africa.

A study conducted in Venda, South Africa (31), revealed that self-employed clients had no problems keeping appointments with their clinics. Those employed faced some challenges in meeting their review dates (31). However, in our study, both self-employed and employed clients missed their appointments, explaining that they had no chance due to work issues. Those employed further outlined that their managers were using a “*No Work, No Pay Policy”.* Another study conducted in Lesotho found that participants, especially men, preferred assistance on weekends to avoid missing work on business days (13). A study conducted in the Eastern Cape, South Africa, found that employers violated patients’ rights by refusing to accept their clinic sick notes after collecting ARVs (27).

Our study revealed that men discontinued on ARVs due to poor working relationships with their healthcare workers. The findings remained consistent with those of other African countries, such as Malawi, Uganda, and South Africa (29–31), where most clients reported that the bad attitudes of healthcare workers prevented them from coming to the facility to access the services. Patient-centred care is essential in improving men’s outcomes. The study conducted in Malawi found that staff burnout leads to negative attitudes among health care workers (29). The same study also found that several African countries are still facing a lack of healthcare workers to provide HIV services, leading to staff burnout and some bad attitudes (29). In these cases, it is crucial to foster good patient-clinician relationships to improve clients’ adherence to ARVs (26).

The study findings reveal that men stop taking their ARVs due to medication side effects. Some participants reported drowsiness as the main side effect. The bad experiences influenced clients to abandon their regimens (29). Similarly, the study conducted in Uganda and in Eswatini on adolescents stated that adolescents stopped taking their medication due to ARV side effects (28, 30). Also, the study conducted in Bloemfontein, South Africa, revealed that ART patients stopped ARVs because they were not coping with the side effects of the ARVs (32).

This study revealed that Christian and cultural beliefs and norms had played a pivotal role in shaping the community and also in how the community perceives the facility, to avoid cultural or religious shock that can be portrayed by health care workers when providing services. It also highlighted how controversial and influential religious and traditional leaders were in shaping and controlling clients’ decisions to attend the clinic, thereby compromising the appointment schedule. The scoping review done in Sub-Saharan Africa among Pentecostal Christians living with HIV found similar findings to our study, as it had shown that the religious beliefs and practises had compromised their commitment to taking their prescribed HIV medication, thus leading to poor viral load suppression and other clinical outcomes (33).

According to this study, some men discontinued their HIV medication during the Covid 19 pandemic, because of the strict facility Covid 19 prevention measures. They highlighted that during the pandemic era, they were not allowed to access the facility without a COVID-19 vaccination card, which hindered some men from receiving their HIV medication on their planned appointment day. A systematic mixed-method study conducted in middle-income countries found that the COVID-19 adverse impact was primarily attributed to a few individual and facility-related factors (34).

The study effectively met all of its objectives, offering a comprehensive understanding of the factors that influence men to disengage from their treatment. First, the study investigated the socio-economic elements that influence men’s discontinuing behaviour. The research revealed that income volatility, employment issues, and financial responsibilities all have a substantial impact on men’s capacity to adhere to commitments or treatments, leading to higher discontinuation rates. These socio-economic issues frequently create barriers that hinder males from consistently participating.

Furthermore, the study examined health system characteristics that led men to discontinue their treatment. The findings showed that access to health services, quality of care, healthcare professionals’ attitudes, and service organisation all have an important effect. For example, long waiting times and perceived stigma in hospital facilities dissuade men from completing treatment or following up, increasing discontinuity rates. The results of this study emphasise the importance of improving the healthcare system to meet men’s unique needs.

Overall, the research findings met the objectives by confirming the complex socio-economic, healthcare, and cultural factors that contribute to men disengaging from their treatment. These findings provide a platform for developing targeted interventions to address the most common reasons for disengagement from care and lower male default rates.

### Strengths

The data was collected by the principal investigator, who was trained in qualitative research methods to reduce data inaccuracies. The data was collected in the quiet consultation room to improve the participants’ natural environment.

### Limitations

Although the study provided valuable insights into factors that influence men to discontinue ARVs in the Maseru Seventh-day Adventist Church in Lesotho, the results are not generalisable due to the study’s design and sample size limitations. Still, the study methods can yield similar results if repeated in a different population setting.

## Conclusion

The findings of this study were that men discontinued on ARVs because they were away from home due to work-related issues in either Lesotho or South Africa. Men are believed to be the source of income (breadwinners) of their households. Therefore, it becomes more difficult for them to access the facility than for their female counterparts. Furthermore, the study has provided essential information on factors that influence men’s discontinuation of treatment, which can inform policy development and prevention strategies. Finally, more research is needed to improve staff-client relationships across different facilities.

## Data Availability

The raw data supporting the conclusions of this article will be made available by the authors, without undue reservation.
